# Bisphenol A Effects on Neurons’ Neurochemical Character in the Urinary Bladder Intramural Ganglia of Domestic Pigs

**DOI:** 10.3390/ijms242316792

**Published:** 2023-11-27

**Authors:** Krystyna Makowska, Piotr Lech, Sławomir Gonkowski

**Affiliations:** 1 Department of Clinical Diagnostics, Faculty of Veterinary Medicine, University of Warmia and Mazury in Olsztyn, Oczapowskiego 14, 10-957 Olsztyn, Poland; 2 Agri Plus sp. Z o.o., Marcelinska Street 92, 60-324 Pozan, Poland; 3 Department of Clinical Physiology, Faculty of Veterinary Medicine, University of Warmia and Mazury in Olsztyn, Oczapowskiego 13, 10-957 Olsztyn, Poland

**Keywords:** endocrine disruptors, excretory system, nervous system, neurochemical coding, bisphenol A

## Abstract

Bisphenol A (BPA), a substance globally used to produce plastics, is part of many everyday items, including bottles, food containers, electronic elements, and others. It may penetrate the environment and living organisms, negatively affecting, among others, the nervous, immune, endocrine, and cardiovascular systems. Knowledge of the impact of BPA on the urinary bladder is extremely scarce. This study investigated the influence of two doses of BPA (0.05 mg/kg body weight (b.w.)/day and 0.5 mg/kg b.w./day) given orally for 28 days on the neurons situated in the ganglia located in the urinary bladder trigone using the typical double immunofluorescence method. In the study, an increase in the percentage of neurons containing substance P (SP), galanin (GAL), a neuronal isoform of nitric oxide synthase (nNOS—used as the marker of nitrergic neurons), and/or cocaine- and amphetamine-regulated transcript (CART) peptide was noted after BPA administration. The severity of these changes depended on the dose of BPA and the type of neuronal factors studied. The most visible changes were noted in the cases of SP- and/or GAL-positive neurons after administering a higher dose of BPA. The results have shown that oral exposure to BPA, lasting even for a short time, affects the intramural neurons in the urinary bladder wall, and changes in the neurochemical characterisation of these neurons may be the first signs of BPA-induced pathological processes in this organ.

## 1. Introduction

Bisphenol A (BPA) is an organic substance synthesized for the first time in the late 19th century by the condensation of acetone and two molecules of phenols [1]. Due to its properties, such as its high thermal stability, plasticity, and relatively cheap synthesis, BPA is commonly used in the plastic industry to produce polycarbonates and epoxy and vinyl ester resins [2]. BPA is a component of many everyday objects, including, among others, bottles, clothes, electronic elements, house equipment, furniture, and office supplies. This compound is also present in thermal paper, paints, and dental fillings [2,3].

It has been found that BPA may be washed out from plastics and penetrate the environment [4]. Up to now, the presence of this compound has been observed in surface water and tap water, air, plants, house dust, and soil around the world [2,4]. Moreover, BPA penetrates human and animal organisms through the digestive tract, respiratory system, and skin [1]. The main route of exposure to BPA is through the digestive system. Therefore, items containing BPA that come into contact with food and drinking water, such as bottles, dishes, cutlery, and containers, are especially dangerous because BPA may penetrate directly into food and then into the digestive tract [1,2].

Previous studies confirm that BPA shows strong endocrine-disrupting properties [1]. It has been shown that BPA negatively affects various internal organs and systems, including, among others, the nervous, reproductive, and endocrine systems, immune cells, digestive tract, and heart [1,2,5,6,7,8,9]. Moreover, some studies have described correlations between the degree of exposure to BPA and the occurrence frequency of diabetes, obesity, hypertension, and even neurodegenerative diseases [1,2,10,11].

However, the negative influences of BPA on various internal systems are relatively unknown. One such system is the excretory system. Previous studies on this topic have mainly focused on changes in the kidneys and have found that BPA causes developmental disorders and renal failure, accompanied by oliguria and albuminuria [6]. It has also been shown that exposure to BPA results in inflammatory processes in the kidneys, tubular injury, and renal fibrosis [12]. In turn, epidemiological studies on humans have found correlations between exposure to BPA and the glomerular filtration rate, urine albumin-to-creatinine ratio, and the risk of chronic kidney disease [13,14].

Information regarding the effects of BPA on the urinary bladder is even more limited. It is known that exposure to BPA may result in inflammatory processes and neoplasms in this organ [15]. Moreover, BPA may contribute to disturbances in urinary voiding, but the exact mechanisms are unclear [16]. It is possible that BPA can cause urinary voiding dysfunction by affecting the nervous structures that supply this organ. This is especially likely as BPA has been shown to negatively impact neuronal cells in other parts of the nervous system, leading to neurodegenerative changes, synaptogenesis disorders, disturbances in calcium metabolism, and disruptions in the production of neuronal active factors [17,18,19,20,21].

Urinary bladder innervation consists of intrinsic and extrinsic innervation. The former comprises the postsynaptic parasympathetic neuronal cells grouped into the ganglia in the urinary bladder wall and supplying the mucosal and muscular layers [22]. Such ganglia have been described in the urinary bladder of humans, guinea pigs, and domestic pigs, and the largest number of them have been found in the urinary bladder trigone—the triangular-shaped region at the base of the bladder limitedly defined by the two ureteral orifices and the internal urethral meatus [23]. The urinary bladder is also innervated by the nerves of external origin, which are projections of the parasympathetic neurons located in the parasympathetic pelvic ganglia, sympathetic neurons located in sympathetic trunks, hypogastric plexuses, inferior mesenteric ganglia, as well as sensory neurons placed in the dorsal root ganglia [22,24,25,26,27].

Previous studies have shown that neurons located in the urinary bladder intramural ganglia are diverse in terms of their neurochemical characterisation and may contain a wide range of active neuronal factors [28,29,30,31]. Moreover, it has been found that these neurons take part in pathological processes within the urinary bladder and may change their neurochemical profile under the impact of various pathological and toxic factors [32,33,34]. However, it should be underlined that knowledge about the impacts of BPA on the urinary bladder intramural neurons is extremely scarce and limited to one study, in which BPA-induced changes in the number of neurons containing vasoactive intestinal polypeptide were found [34].

Considering the above, the present investigation aimed to evaluate (for the first time) the influence of two various doses of BPA on neurons located in the urinary bladder intramural ganglia and containing neuronal factors, such as substance P (SP), galanin (GAL), a neuronal isoform of nitric oxide synthase (nNOS—used as a marker of nitrergic neurons), and the cocaine- and amphetamine-regulated transcript (CART) peptide, which are known to play important roles in the regulation of the urinary bladder functions [29,30,35,36,37]. The obtained results will allow for a better understanding of the harmful effects of BPA on the functioning of the bladder. Moreover, due to the considerable similarity between the organisation of the intramural ganglia of the urinary bladder in humans and domestic pigs [38,39], the present study may be the first step in understanding the processes connected with BPA’s impact on the human urinary bladder.

## 2. Results

During the present study, neurons immunoreactive to all active substances included in the study were observed in the ganglia within the urinary bladder wall both in physiological conditions and after BPA administration (Table 1). In the control pigs, the largest group of neurons were cells containing nNOS, the percentage of which was 43.72 ± 1.20% of all PGP 9.5-positive cells. Neurons immunoreactive to CART were slightly less numerous. Their percentage amounted to 38.65 ± 0.30%. In turn, populations of neurons immunoreactive to GAL and/or SP were much less numerous. The percentage was 26.16 ± 0.74% for GAL–positive cells and 20.70 ± 0.82% for neurons containing SP.

**Figure 1:**
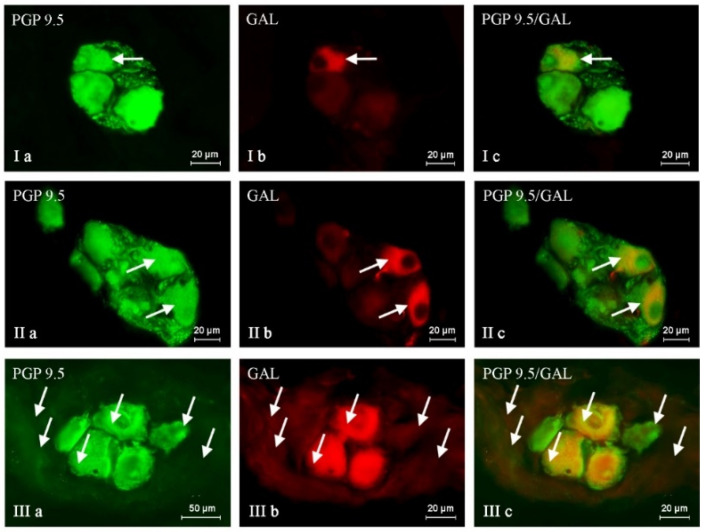
Nerve cells immunoreactive to protein gene product (PGP 9.5; used here as a pan-neuronal marker) (**a**) and galanin (GAL) (**b**) in the intrinsic ganglia of the porcine urinary bladder trigone wall under the physiological condition (I), and shown after administration of low (II) and high (III) doses of BPA. GAL-positive neurons are marked with arrows. The right column (**c**) was created by overlapping pictures (**a**) and (**b**).

**Figure 2:**
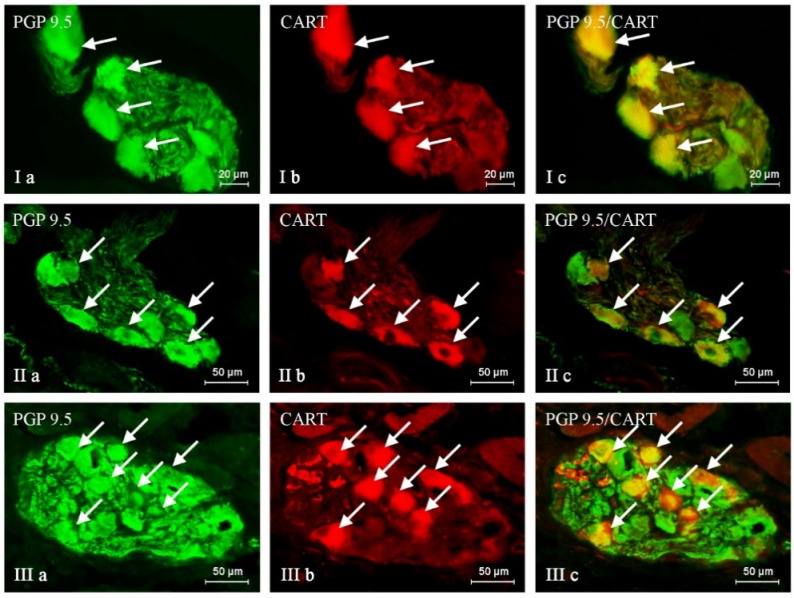
Nerve cells immunoreactive to protein gene product (PGP 9.5; used here as a pan-neuronal marker) (**a**) and cocaine- and amphetamine-regulated transcript (CART) peptide (**b**) in the intrinsic ganglia of the porcine urinary bladder trigone wall under the physiological condition (I), and after administration of low (II) and high (III) doses of BPA. CART-positive neurons are marked with arrows. The right column (**c**) was created by overlapping pictures (**a**) and (**b**).

**Figure 3:**
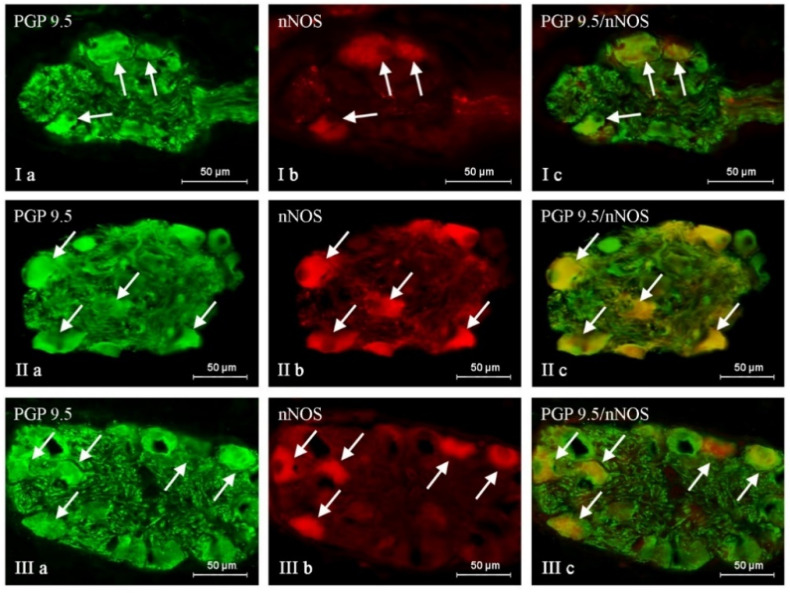
Nerve cells immunoreactive to protein gene product (PGP 9.5; used here as a pan-neuronal marker) (**a**) and neuronal isoform of nitric oxide synthase (nNOS—used as the marker of nitrergic neurons) (**b**) in the intrinsic ganglia of the porcine urinary bladder trigone wall under the physiological condition (I), and after administration of low (II) and high (III) doses of BPA. nNOS-positive neurons are marked with arrows. The right column (**c**) was created by overlapping pictures (**a**) and (**b**).

**Figure 4:**
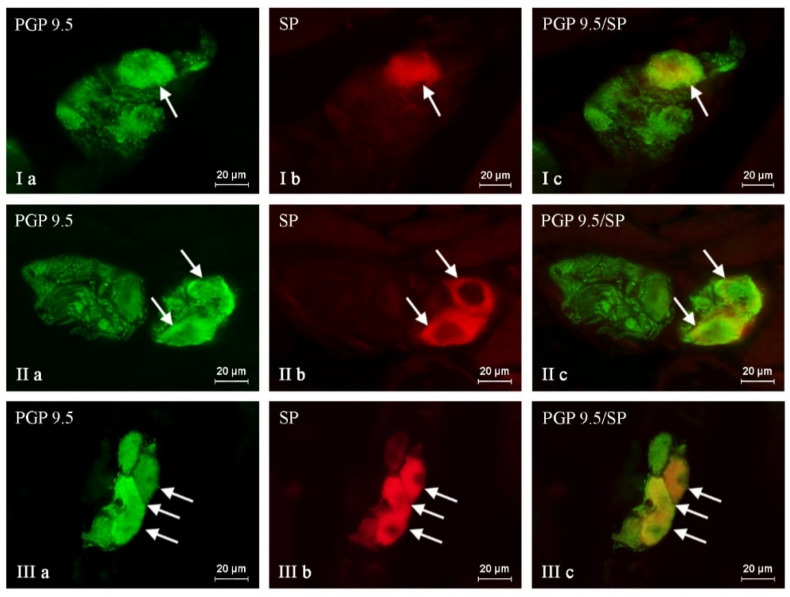
Nerve cells immunoreactive to protein gene product (PGP 9.5; used here as a pan-neuronal marker) (**a**) and substance P (SP) (**b**) in the intrinsic ganglia of the porcine urinary bladder trigone wall under the physiological condition (I), and after administration of low (II) and high (III) doses of BPA. SP-positive neurons are marked with arrows. The right column (**c**) was created by overlapping pictures (**a**,**b**).

The administration of BPA generally caused an increase in the percentage of neuronal cells containing all active substances studied, and the severity of the changes depended on the type of neuronal factor and the BPA dose (Table 1, Figure 1, Figure 2 and Figure 3). After the administration of a lower BPA dose, the most visible changes were noted in GAL-positive neurons (Figure 1). Their percentage in the BPA 1 group accounted for 35.29 ± 1.16% of all PGP 9.5-positive cells (an increase of about 9 percentage points (p.p.) compared to the control group) (Table 1 and Table 2). The second most visible changes after the administration of the lower dose of BPA were found in the population of neurons containing CART (Figure 2). Their percentage increased by about 6 p.p. and achieved 44.95 ± 0.57% (Table 1 and Table 2). In the BPA 1 group, similar to the control animals, the most numerous populations were neurons containing nNOS, whose percentage was 47.49 ± 0.30% (Table 1, Figure 3). However, the impact of the lower doses of BPA on this population was relatively small because the increase in the percentage of nNOS-positive cells in the BPA 1 group compared to the control pigs accounted for 4 p.p (Table 2). Even less-pronounced changes were observed in the populations of SP-immunoreactive neurons, whose percentage was 23.86 ± 0.40% (an increase of about 3 p.p. compared to the control group) (Table 2, Figure 4).

Higher doses of BPA caused more pronounced changes in the size of the neuronal populations studied. The most visible increase in percentage was noted in the cases of neurons containing SP and/or GAL (Table 1 and Table 2, Figure 1 and Figure 4). The percentage of SP-positive neurons in the BPA 2 group amounted to 34.83 ± 0.64% of all PGP 9.5-immunoreactive cells (an increase of about 14 p.p. compared to the control group) (Table 1 and Table 2). Similar changes (an increase of about 13 p.p.) were observed in neurons containing GAL, the percentage of which in the BPA 2 group amounted to 39.57 ± 0.48% (Table 1 and Table 2). The visible changes in animals in the BPA 2 group also concerned neuronal cells containing CART (Figure 2). The percentage of such neurons amounted to 49.05 ± 1.70%, and compared to the control group, it was higher by 11 p.p (Table 1 and Table 2). The population of nNOS-immunoreactive neuronal cells was the most numerous in the BPA 2 group, but the percentage of such neurons was the least susceptible to changes under the influence of BPA (Figure 3). In particular, the percentage of nNOS-positive neurons was 49.60 ± 1.08% and was only 6 p.p. higher than the value observed in the control animals (Table 1 and Table 2). The summarization of the changes in p.p. noted in the present study is presented in Table 2.

## 3. Discussion

The results obtained in this study have shown the presence of all studied substances in the neurons located in the intramural ganglia, which is in agreement with previous observations describing these substances in the intramural urinary bladder neurons in various mammal species, including humans [30,31,34,35,36,37,40,41,42,43,44,45]. Conversely, differences concerning the sizes of neuronal populations that are immunoreactive to particular neuronal factors between the present study and previous studies conducted on domestic pigs are clearly visible. In particular, this concerns neurons containing nNOS and CART, the number of which observed in previous studies was significantly smaller than that noted in the present investigation [35,36,40,44]. These discrepancies may result from environmental factors affecting the animals or some differences in methodology, but could also result from interracial differences (in previous studies, the pigs of the Large White Polish breed were investigated) [30,31,40,42,43]. However, the results obtained in the present study are similar to those noted in observations conducted on the human urinary bladder [28], which, together with other studies on the similarities in organisation between human and porcine nervous systems [40], confirms that the domestic pig seems to be a good animal model for studies concerning processes occurring in human neuronal cells.

The presence of the neuronal factors investigated in a relatively high number of neurons strongly suggests the important roles of these substances in regulating urinary bladder functions. This is also reflected in previous studies. It is known that nitric oxide (nitrergic neurons containing nNOS were the most numerous population of neuronal cells studied in the present experiment) affects the activity of muscles located in the urinary bladder wall [36] and regulates intramural blood flow through a relaxant effect on the blood vessels [41]. Moreover, nitric oxide takes part in the conduction of sensory stimuli from the urinary bladder, as evidenced by the presence of nNOS in neurons supplying this organ [40]. In turn, GAL inhibits bladder emptying, probably by activating mechanisms related to opioid substances [37], and SP activates the urinary bladder muscles, increasing the frequency of urination via a simultaneous reduction of its volume [45]. SP and GAL are responsible for relaying sensory information from the urinary bladder to the central nervous system. The role of CART in regulating urinary bladder activity is not well understood, but it is believed to play a role in the growth and maturation of cells within the bladder during development. There is a high likelihood that CART also affects the muscles of the urinary bladder [35,44].

In light of previous studies, it is also known that substances included in this investigation take part in the pathological reactions within the urinary bladder caused by inflammatory processes and toxic factors [40,41,42,43,44]. The current study also confirmed this. However, due to the multidirectional influence of BPA on living organisms and the various reasons for changes in the neurochemical characterisation of neurons, the exact reasons for the obtained results are difficult to explain at the current research stage. In particular, changes noted in the present study may result not only from the increase in the synthesis of neuronal factors on a transcriptional or translational level, or the BPA-induced changes in mRNA or DNA known from previous studies [46,47], but also from the inhibition of the transport of neuronal factors from the cell body to the nerve endings.

The observed changes may result from the direct impact of BPA on the nervous system. Previous studies have reported that BPA shows neurodegenerative properties, appearing as a result of damage to the development of synapses and neurites [48], disturbances in calcium ions’ transport through neuronal cell membranes [49], and mitochondrial failure [50]. Therefore, the changes in the neurochemical characterisation of neurons noted in the present study may be the organism’s response to the neurodegenerative actions of BPA aimed at the neuroprotective processes and maintenance of homeostasis in the nervous system. It is likely that all neuronal factors included in the present study have been described as substances with neuroprotective and antidegenerative properties, both in the central and peripheral nervous system [51,52,53,54,55,56].

The next reason for the observed changes may be connected to the relatively well-known pro-inflammatory activity of BPA and its influence on the immune system [50]. This influence may result in fluctuations in the levels of factors modulating immune cell activity, and it should be underlined that all the neuronal substances studied in the present experiment show such properties. SP is known as the most well-known factor modulating the activity of immune system cells. It stimulates the secretion of pro-inflammatory substances, such as IL-1, IL -6, and TNF-α [57], simultaneously increasing phagocytic activity and histamine release [58,59]. Studies conducted on the urinary bladder have shown that inflammatory processes significantly increase the concentration of SP within this organ, and this phenomenon is accompanied by an increase in the levels of pro-inflammatory cytokines, mast cell degranulation, and lymphocytic infiltration [45]. GAL and nitric oxide are also involved in modulating immune cell activity. GAL, among others, causes an increase in the production of proinflammatory cytokines (mainly IFN-γ) [60] and decreases the synthesis of TNF α [61]. In turn, nitric oxide is one of the most important immunomodulatory factors affecting many processes within various immune cells [62]. Moreover, CART, whose immune system function is unclear, has immunomodulatory activity but, in contrast to SP, causes a reduction in the synthesis and release of pro-inflammatory factors with a simultaneous increase in the activity of anti-inflammatory cytokines [52].

Interestingly, BPA administration caused similar changes (an increase in number) in populations of neurons containing these factors, which have an opposite effect. The reason for such a phenomenon is unclear, but a similar situation has been noted under the impact of BPA within intestinal innervation [63,64]. It should be underlined that the BPA doses used in the present study were relatively low, and they did not cause visible inflammatory symptoms. Admittedly it cannot be excluded that the fluctuations in the levels of neuronal factors noted in the present study may be the first signs of a subclinical inflammatory state, but it is important to stress that this thesis is only a guess and its confirmation is not possible without further comprehensive molecular and/or biochemical research

The third reason for the observed changes may be connected with the impact of BPA on muscular contraction. Although the impacts of BPA on the urinary bladder muscles has not yet been studied, it is known that this substance inhibits the amplitude and frequency of contractions of muscles located in the wall of the intestine and uterus of various mammal species [65,66,67,68,69]. Therefore, similar effects of BPA on the urinary bladder are likely. The mechanisms of such relaxant activities of BPA are not clear. Previous studies suggest various pathways involved in the BPA-induced inhibition of muscular contraction, including the oxytocin- and prostaglandin-related pathway [70], norepinephrine-dependent conduction [71], and/or the nitric oxide-dependent pathway [66]. In light of the present study, where the number of nNOS–positive cells increased after BPA administration with a simultaneous increase in the number of neurons containing the other neuronal substances, which are known to be co-transmitters and/or neuromodulators in nitrergic neurons [72], the possibility of the occurrence of the nitric oxide-dependent pathway in the urinary bladder seems probable.

This study provides evidence that BPA could impact the nerve supply of the urinary bladder. However, it remains unclear whether the amounts of BPA that humans and animals are exposed to in their daily lives could lead to similar changes in nerve supply. In light of this, the European Union’s lower dose of BPA was used in this study (0.05 mg/kg b.w./day), which for many years was considered a tolerable daily intake (TDI), i.e., a safe dose that should not cause any changes in the human body [73]. The TDI for BPA was reduced from 5 to 4 µg/kg b.w./day in 2015, and a proposal for a further reduction exists [74]. Simultaneously, the dose of 0.05 mg/kg b.w./day is still a TDI in some countries outside the European Union [75], although it is not completely neutral for living organisms, which has been confirmed by previous studies [20,34,63,64] and the present investigation. It should be pointed out that the BPA doses used in the present study are higher than the doses to which living organisms are usually exposed in “everyday life” [2]. However, it is known that humans in some situations may be exposed to higher doses of BPA, which is connected with natural environment pollution, particular jobs, or other environmental factors [2,3]. For example, persons with numerous old-type dental fillings can be exposed to BPA in doses as high as 30 mg/day [76]. Moreover, it should be underlined that during the present study, BPA was administered relatively quickly. Considering the widespread exposure of humans and animals to BPA [77], it cannot be excluded that long exposure to even lower doses of this substance would result in changes in neuronal neurochemical profiles.

The present study (for the first time) has shown that BPA affects the neurochemical character of various classes of neurons in the intramural ganglia in the porcine urinary bladder. The observed changes depend on the BPA dose and type of neuronal factor studied. The changes in the populations of various neurochemical classes of neurons confirm the multidirectional impact of BPA on the nervous system. The influence of BPA on the neurons in the urinary bladder wall is visible under the impact of relatively low doses of this substance given for a short period (28 days). The observed changes may result from the neurodegenerative, inflammatory, and/or muscle relaxant properties of BPA. However, elucidating the exact mechanisms of the BPA-induced fluctuations in the neurochemical profile of the nervous structures innervating the urinary bladder, and the effects of such fluctuations on the functioning of this organ requires further comprehensive research.

## 4. Materials and Methods

The investigation was conducted on 15 female pigs of the Piétrain × Duroc breed. The animals were eight weeks old and weighed about 18–20 kg. During the experiment, the animals were kept in standard conditions in the animal house of the Faculty of Veterinary Medicine at the University of Warmia and Mazury in Olsztyn (Poland). The pigs were held in playpens (five animals in each) and fed twice a day with commercial feedstuff suitable for their animal age and species. Moreover, the animals had constant access to water from mechanical drinkers. All activities within the framework of the experiment were approved by the Local Ethics Committee Responsible for the Experimental Animals in Olsztyn (Poland) (decision numbers 28/2013 of 22 May 2013 and 65/2013/DLZ of 27 November 2013).

After transport, the pigs were randomly divided into three groups—five animals in each group and subjected to a five-day adaptation period. Each group was placed in a separate playpen. The groups of animals included in the experiment were as follows: (1) the control (C) group—in which the animals received orally empty gelatine capsules; (2) BPA 1 group—in which the pigs received capsules with BPA (>99%, catalogue no: 239658-250G, Sigma-Aldrich, Poznan, Poland) at a dose of 0.05 mg/kg body weight (b.w.)./day; (3) BPA 2 group—in which the pigs received capsules with BPA at a dose of 0.5 mg/kg b.w./day. In all groups, the capsules were administered in the same manner before the morning feeding. The animals were weighed every week to establish the exact dose of BPA. All animals were euthanised after 28 days of BPA administration. To this aim, the pigs were premedicated with Stresnil (Janssen, Beerse, Belgium, 75 μL/kg of b.w., given intramuscularly) and after 20 min, they were euthanised with an overdose of sodium thiopental (Thiopental, Sandoz, Kundl, Austria, given intravenously through the marginal ear vein)

Immediately after the death of the animals, the urinary bladder trigones were collected. Tissues were fixed in 4% buffered paraformaldehyde (pH 7.4) for one hour and rinsed in a phosphate buffer for three days with daily buffer exchange. Fragments of the urinary bladder were then put into 18% phosphate-buffered sucrose and stored at 4 °C for at least three weeks. After this period, tissues were frozen at −22 °C, cut into 14 μm thick sections with a cryostat (HM 525, Microm International, Dreieich, Germany), mounted on microscopic slides, and stored at −18 °C until further study.

The fragments of the urinary bladder were studied using the double immunofluorescence technique according to the method previously described by Makowska et al. in 2021 [34]. In brief, the study was performed according to the following scheme (all stages were performed at room temperature): (1) After their removal from the freezer, the slides with urinary bladder trigone fragments were dried for one hour; (2) The tissues were incubated with “blocking” solution (10% normal goat serum, 0.1% bovine serum albumin, 0.01% NaN_3_, 0.25% Triton x-100, and 0.05% thimerosal in phosphate-buffered saline—PBS) to eliminate non-specific labelling; (3) Urinary bladder fragments were incubated overnight in a humidity chamber with the mixture of two primary antibodies, one of which was directed against the pan-neuronal marker protein gene product 9.5 (PGP 9.5) and the other against one of the following substances: SP, GAL, nNOS, or CART. The specifications of the antibodies are presented in Table 3; (4) The tissues were incubated with species-specific secondary antibodies conjugated with fluorochromes (Table 3) for 1 h for visibility of the complexes “antigen-primary antibody”; (5) The urinary bladder fragments were covered with buffered glycerol and cover slides. After each step of labelling, the slides with the urinary bladder fragments were rinsed in PBS for 30 min with a PBS exchange every 10 min. In order to check the specificity of the labelling, typical tests of antibodies were conducted. These tests included the pre-absorption of the antisera with an appropriate antigen, omission, and the replacement of primary antisera by non-immune sera. All of the above tests yielded negative results which eliminated the nonspecific staining.

The labelled urinary bladder fragments were viewed under an Olympus BX51 microscope (Olympus, Tokyo, Japan) equipped with epi-fluorescence and the appropriate filter sets. To determine the percentage of neuronal cells containing particular neuronal factors, at least 500 cells immunoreactive to PGP 9.5 were evaluated for each substance included in the study. Only double-labelled neurons with a visible nucleus were considered, and the number of PGP 9.5-positive cells included in the study was considered to be 100%. To rule out the double counting of the same cells, the urinary bladder sections subjected to evaluation were located at least 50 μm apart from each other. The obtained results were pooled and presented as mean ± Standard Error of the Mean (SEM).

Our statistical analysis was completed via an Anova test (Statistica 13, StatSoft, Inc., Cracow, Poland), and differences were considered statistically significant at *p* ≤ 0.05.

## Figures and Tables

**Table 1:** The percentage (%) of neurons immunoreactive to pan-neuronal marker protein gene product 9.5 (PGP 9.5) which was simultaneously immunoreactive to substance P (SP), galanin (GAL), neuronal isoform of nitric oxide synthase (nNOS), and/or cocaine- and amphetamine-regulated transcript (CART) peptide in the wall of urinary bladder trigone in the control group (C), the experimental group 1 (BPA 1), and experimental group 2 (BPA 2). Data expressed as mean% ± SEM. The results were considered statistically significant at *p* < 0.05 and are marked with *.

	PGP/SP	PGP/GAL	PGP/nNOS	PGP/CART
C	20.70 ± 0.82	26.16 ± 0.74	43.72 ± 1.20	38.65 ± 0.30
BPA 1	23.86 ± 0.40 *	35.29 ± 1.16 *	47.49 ± 0.30 *	44.95 ± 0.57 *
BPA 2	34.83 ± 0.64 *	39.57 ± 0.48 *	49.60 ± 1.08 *	49.05 ± 1.70 *

**Table 2:** The increase of the percentage of neurons containing pan-neuronal marker protein gene product 9.5 (PGP 9.5) that were simultaneously immunoreactive to substance P (SP), galanin (GAL), neuronal isoform of nitric oxide synthase, and/or cocaine- and amphetamine-regulated transcript (CART) peptide in the wall of urinary bladder trigone given in the percentage points (p.p.) in both experimental groups (BPA 1 and BPA 2) in comparison to the control group (in control animals, level 0 was assumed).

	PGP/SP	PGP/GAL	PGP/nNOS	PGP/CART
BPA 1	+3.16	+9.13	+3.77	+6.3
BPA 2	+14.13	+13.41	+5.88	+10.4

**Table 3:** List of antisera and reagents used in immunohistochemical investigations.

Primary Antibodies				
Antigen	Code	Species	Working Dilution	Supplier
PGP 9,5	7863-2004	Mouse	1:1000	Biogenesis Ltd., Poole, UK
SP	8450-0505	Rat	1:1000	Bio-Rad (AbD Serotec), Kidlington, UK
CART	1-003-61	Rabbit	1:8000	Phoenix Pharmaceuticals, INC, Belmont, CA, USA
GAL	T-5036	Guinea Pig	1:2000	Peninsula, New York, NY, USA
nNOS	AB5380	Rabbit	1:6000	MercMillipore, DEU, İzmir, Turkey
Secondary antibodies				
Reagents			Working dilution	Supplier
Alexa fluor 488 donkey anti-mouse IgG			1:1000	Invitrogen, Carlsbad, CA, USA
Alexa fluor 546 donkey anti-rabbit IgG			1:1000	Invitrogen
Alexa fluor 546 donkey anti-rat IgG			1:1000	Invitrogen
Alexa fluor 546 donkey anti-guinea pig IgG			1:1000	Invitrogen

## Data Availability

The datasets generated and/or analyzed during the current study are available from the corresponding author upon reasonable request.
